# General Hydrate Formation Temperature Model for CO_2_, H_2_S, Pure Hydrocarbon Gases, and Sweet and Sour Gas Mixtures with or without Common Salts and Organic Inhibitors

**DOI:** 10.1021/acsomega.6c01670

**Published:** 2026-05-13

**Authors:** Eissa M. El-M. Shokir, Azza El-S. B. Ibrahim, Mostafa M. Abdelhafiz, Ali E. Shokir

**Affiliations:** † Petroleum Engineering Department, Faculty of Engineering, Cairo University, Giza 12613, Egypt; ‡ 230798 Electronics Research Institute (ERI), El Nozha, Cairo 12622, Egypt; § Institute of Applied Mechanics, 26534 TU Clausthal, Clausthal-Zellerfeld 38678, Germany; ∥ Mechanical Engineering Department, Faculty of Engineering, 110148 Cairo University, Giza 12613, Egypt

## Abstract

Gas hydrate formation conditions are a critical problem in the production, processing, and transportation of natural gases. The precise determination of the hydrate formation pressure and temperature is essential. The experimental determination of hydrate formation pressure and temperature is a time-consuming and costly process. Therefore, many published mathematical and thermodynamic models exist to estimate the hydrate formation temperature (HFT). However, most of these models lack generalization, exhibit low accuracy, and have limited application to complex gas systems, including gas hydrate formers and nonhydrocarbon gases without mixed salts and organic inhibitors. This paper presents a generalized HFT model for CO_2_, H_2_S, pure hydrocarbon gases, sweet gas mixtures, and sour gas mixtures in the presence or absence of different salts and organic inhibitors (NaCl, MgCl_2_, KCl, methanol, acetone, ethylene glycol, isopropyl alcohol, 2-pyrrolidone formamide, and *N*,*N*-dimethylformamide) using alternating conditional expectation (ACE) algorithms. The blind test of the new model yielded a perfect match with the measured HFT and demonstrated superiority over earlier HFT-published models for hydrocarbon gases, sweet gas mixtures, and sour gas mixtures.

## Graphical abstract

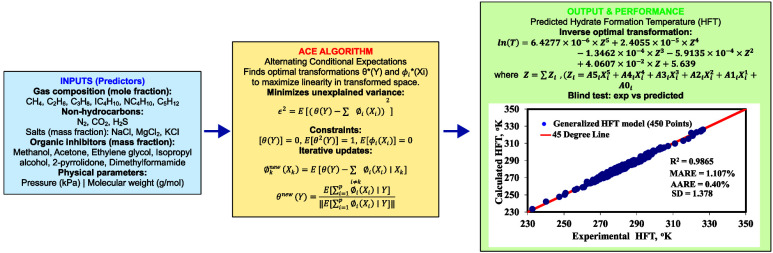

## 1 Introduction

In petroleum exploration and production operations, gas hydrates are a very costly problem, because they block the hydrocarbon flow and expose persons to hazards.^1,2^ Therefore, accurate determination of hydrate equilibrium conditions is vital for designing gas production and transportation systems in safe operating conditions. Usually, inhibitors are added to processing lines to inhibit the formation of hydrates. The most common gas hydrate inhibitors are organic inhibitors such as methanol, ethylene glycol, triethylene glycol, and salts such as sodium chloride (NaCl), potassium chloride (KCl), or calcium chloride (CaCl_2_), and magnesium chloride (MgCl_2_) have inhibiting effects.^3^

Due to the time-consuming and expensive nature of experimental studies on gas hydrate conditions, as well as the complex application of the thermodynamic models,^4–7^ several mathematical correlations have been published to determine the hydrate equilibrium conditions.^8–11^

In 1942, Carson and Katz^12^ proposed the K-value method for predicting the hydrate formation conditions using empirically estimated equilibrium constants. In 1945, Katz^13^ generated charts relating the hydrate pressure and temperature with gas gravity, and this method was named the gas gravity method. Alternatively, many authors have used the regression analysis method to correlate the hydrate equilibrium conditions as a function of gas gravity.^14–25^ Most of these correlations have low accuracy in calculated hydrate conditions for different gas mixtures having equal specific gravities. Other published correlations correlated the temperature of gas hydrate formation with the gas molecular weight (MW) and pressure.^25,26^

Abooali and Khamehchi,^27^ Soroush et al.,^28^ and Mesbah et al.^29^ proposed three models based on genetic programming, artificial neural network (ANN), and least-squares support vector machine (LSSVM), respectively, for estimating the gas hydrate-formation temperature as a function of pressure and gas gravity or gas molecular weight (*M*_w_). Additionally, these published correlations predicted the gas hydrate temperature or pressure for the pure gases or gas mixtures but lacked information on the hydrate inhibition.

This paper presents a new generalized HFT model for CO_2_, H_2_S, pure hydrocarbon gases, sweet, and sour gas mixtures in the presence or absence of different salts and organic inhibitors using the alternating conditional expectation (ACE) algorithm. This model is based on 1228 published experimental data sets^2–7,12–35^ of gas hydrate formation temperatures with or without inhibitors. This data set was randomly divided into two groups: the first one contains 778 data points for building the new model; the second group contains 450 data points for blind testing of the developed new model. It contains samples of pure CO_2_, H_2_S, hydrocarbon gases, and gas mixtures including methane, ethane, propane, i-butane, *n*-butane, *n*-pentane, nitrogen, carbon dioxide, and hydrogen sulfide in the presence or absence of different salts and organic inhibitors (NaCl, MgCl_2_, KCl, methanol (CH_4_O), acetone (C_3_H_6_O), ethylene glycol (C_2_H_6_O_2_), isopropyl alcohol (C_3_H_8_O), 2-pyrrolidone formamide (C_5_H_9_NO), and *N,N*-dimethylformamide (C_3_H_7_NO)).

## 2 ACE Algorithm

Traditional linear regression models assume a linear relationship between the predictors and the response. The Alternating Conditional Expectations (ACE) algorithm, introduced by Breiman and Friedman^30^ provides a flexible, nonparametric approach that generalizes this concept. Instead of fitting a model in the original variable space, ACE seeks to find optimal transformations for both the response and predictor variables such that the relationship in this transformed space is as linear as possible.

The fundamental model underlying ACE is expressed as

1 $$\theta \left(Y\right)=\overset{p}{\underset{i=1}{\sum }}\phi_{i}\left(X_{i}\right)+\varepsilon$$

where *Y* is the response variable, *X*_1_, *X*_2_,···, *X*_*p*_ are the predictor variables, θ(·) is a transformation applied to the response variable *Y*, ϕ_*i*_(·) are transformations applied to each predictor variable *X*_*i*_, and *ε* represents the error term. The goal of ACE is not to estimate a single high-dimensional function, but to estimate *p* + 1 separate one-dimensional functions, θ, ϕ_1_,···,ϕ_*p*_, that maximize the linear association between the transformed variables.^30,31,36^

### 2.1 The Objective: Minimizing Unexplained Variance

The core objective of the ACE algorithm is to identify transformations that minimize the unexplained variance (the error variance) of the linear relationship in the transformed space. This is formalized by minimizing the mean squared error:^30,31,36^

2 $$\epsilon^{2}\left(\theta ,\phi_{1},...,\phi_{p}\right)=E\left[{\left(\theta \left(Y\right)-\sum_{i=1}^{p}\phi_{i}\left(X_{i}\right)\right)}^{2}\right]$$

To ensure identifiability and avoid trivial solutions (like all transformations being zero), the following constraints are typically imposed:

(1)
E[θ(Y)]=0 (the transformed response is centered).
(2)
$E\left[\theta^{2}\left(Y\right)\right]=1$ (the transformed response has unit variance).
(3)
$E\left[\phi_{i}\left(X_{i}\right)\right]=0$ for *i* = 1,···,*p* (the transformed predictors are centered).

### 2.2 The Iterative Algorithm

The ACE algorithm employs an iterative procedure that alternates between updating the response transformation and the predictor transformations, each time conditioning on the current estimates of the others. The process begins with initial arbitrary transformations (e.g., zero-mean functions) and iteratively refines them.

**Step 1: Initialize.**

- Set initial transformations for the predictors, ϕ_1_(*X*_1_),···,ϕ_*p*_(*X*_*p*_). A common choice is to start with centered versions of the original variables.
- Set the initial response transformation, θ­(*Y*), such that the constraints E[θ(Y)]=0 and $E\left[\theta^{2}\left(Y\right)\right]=1$ are satisfied.

**Step 2: Update the Predictor Transformations.** Holding the current θ­(*Y*) and all other ϕ_*j*_ (*j* ≠ *k*) fixed, update each ϕ_*k*_(*X*_*k*_) one at a time. The optimal update is given by the conditional expectation:^30,31,36^

3 $$\phi_{k}^{\mathrm{new}}\left(X_{k}\right)=E\left[\theta \left(Y\right)-\underset{i\neq k}{\sum }\phi_{i}\left(X_{i}\right)|X_{k}\right]$$

This step “extracts” the part of the current transformed response that can be explained by *X*_*k*_, given the contributions of all other predictors.

**Step 3: Update the Response Transformation.** Holding all the updated predictor transformations ϕ_1_(*X*_1_),···,ϕ_*p*_(*X*_*p*_) fixed, update the response transformation. The optimal update is

4 $$\theta^{\mathrm{new}}\left(Y\right)=\frac{E\left[\overset{p}{\underset{i=1}{\sum }}\phi_{i}\left(X_{i}\right)|Y\right]}{∥E\left[\overset{p}{\underset{i=1}{\sum }}\phi_{i}\left(X_{i}\right)|Y\right]∥}$$

The denominator, which is the norm of the conditional expectation, ensures the constraint $E\left[{\left(\theta^{\mathrm{new}}\left(Y\right)\right)}^{2}\right]=1$ is maintained.

**Step 4: Iterate to Convergence.** Steps 2 and 3 are repeated iteratively until the set of transformations stabilizes and the error variance ϵ^2^ no longer decreases significantly.

### 2.3 Output and Interpretation

Upon convergence, the algorithm produces the optimal transformations:

- θ*­(*Y*) for the response.
-
$\phi_{1}^{*}\left(X_{1}\right),...,\phi_{p}^{*}\left(X_{p}\right)$ for the predictors.

In this optimally transformed space, the model is

5 $$\theta^{*}\left(Y\right)=\overset{p}{\underset{i=1}{\sum }}\phi_{i}^{*}\left(X_{i}\right)+e^{*}$$

where e* is the residual error. The quality of the fit is often measured by the maximum multiple correlation coefficient ρ*, which relates to the minimized error variance as (e*)^2^ = 1 – (ρ*)^2^. A value of ρ* close to 1 indicates a strong linear relationship has been found in the transformed space.

## 3 Results

### 3.1 Development of the New Generalized Hydrate Formation Temperature Model

The ACE algorithm was employed to develop a robust new generalized model for predicting the hydrate formation temperature (HFT) for CO_2_, H_2_S, hydrocarbon gases, sweet gas mixtures, and sour gas mixtures in the presence or absence of salts and organic hydrate inhibitors.

The ACE algorithm optimizes the transformations of both the dependent variable (HFT) and multiple independent variables (pressure, gas molecular weight, the mole fraction of the hydrocarbon gases or gas mixtures, the nonhydrocarbon gases, and the mass fraction of the common salts (NaCl, MgCl_2_, KCl), and organic inhibitors (methanol (CH_4_O), acetone (C_3_H_6_O), ethylene glycol (C_2_H_6_O_2_), isopropyl alcohol (C_3_H_8_O), 2-pyrrolidone formamide (C_5_H_9_NO), and *N,N*-dimethylformamide (C_3_H_7_NO))) to achieve a maximal linear correlation between them.

The complete data set of 1228 experimental points^33–35,37–47^ was split into a training set (778 points, 63.4%) and a blind test set (450 points, 36.6%) using stratified random sampling. Stratification was based on gas type and inhibitor presence to maintain representative distributions. This approach follows established data splitting practices^48^ to prevent overfitting and ensure unbiased performance estimates. Random assignment of data points to each subset is consistent with standard practice in machine learning and predictive modeling. The blind test set was used for testing the developed new model and for comparing its results with early published mathematical correlations. Table 1 summarizes the minimum, average, and maximum values of the input and output variables used for building and testing the new model. Moreover, the compiled data set was duplicated by matching gas composition (±0.0001 mole fraction), inhibitor type/concentration (±0.00005 mass fraction), and pressure (±0.05%). Conflicting data were resolved hierarchically: (1) most recent source; (2) isochoric pressure search method; (3) arithmetic mean. Outlier detection (leverage) excluded no points. The final data set contains 1228 unique and consistent points.

**Table 1:** Minimum, Average, and Maximum Values of the Input and Output Variables Used for Building and Testing the New Model

	Value		
Variable	Min	Mean	Max
Hydrocarbon components (mole fraction)			
CH_4_	0.0	0.531	1.0
C_2_H_6_	0.0	0.0791	1.0
C_3_H_8_	0.0	0.0591	0.8
iC_4_H_10_	0.0	0.0191	1.0
nC_4_H_10_	0.0	0.0049	0.15
C_5_H_12_	0.0	0.0005	0.014
Nonhydrocarbon components (mole fraction)			
N_2_	0.0	0.021	0.98
CO_2_	0.0	0.272	1.0
H_2_S	0.0	0.014	1.0
Salts (mass fraction)			
NaCl	0.0	0.0038	0.2
MgCl_2_	0.0	0.0015	0.15
KCl	0.0	0.00052	0.1
Organic inhibitors (mass fraction)			
Methanol (CH_4_O)	0.0	0.0248	0.5
Acetone (C_3_H_6_O)	0.0	0.0069	0.7364
Ethylene glycol (C_2_H_6_O_2_)	0.0	0.0113	0.5
Isopropyl alcohol (C_3_H_8_O)	0.0	0.0021	0.25
*N*-methyl pyrrolidone (C_5_H_9_NO)	0.0	0.0022	0.2
*N,N*-dimethylformamide (C_3_H_7_NO)	0.0	0.0042	0.35
Other variables			
Temperature, *T* (K)	232.55	281.27	330.45
Molecular weight, *M*_*w*_ (g/mol)	16.04	27.95	55.2
Pressure (kPa)	144.8	24,716	1,524,993

The modeling was conducted using the GRACE graphical user interface program,^49^ which implements the ACE framework. The optimal result of the ACE procedure is visualized in Figure 1, which plots the optimally transformed HFT, θ*­(HFT), against the sum of the optimally transformed independent variables. The high degree of linearity in this plot confirms that the ACE algorithm successfully identified a relationship of the form

6 $$\theta^{*}\left(\mathrm{HFT}\right)=\underset{i}{\sum }\phi_{i}^{*}\left(X_{i}\right)$$

where θ* is the optimal transformation for the HFT, and the $\phi_{i}^{*}$ are the optimal transformations for the predictor variables *X*_*i*_.

**Figure 1:**
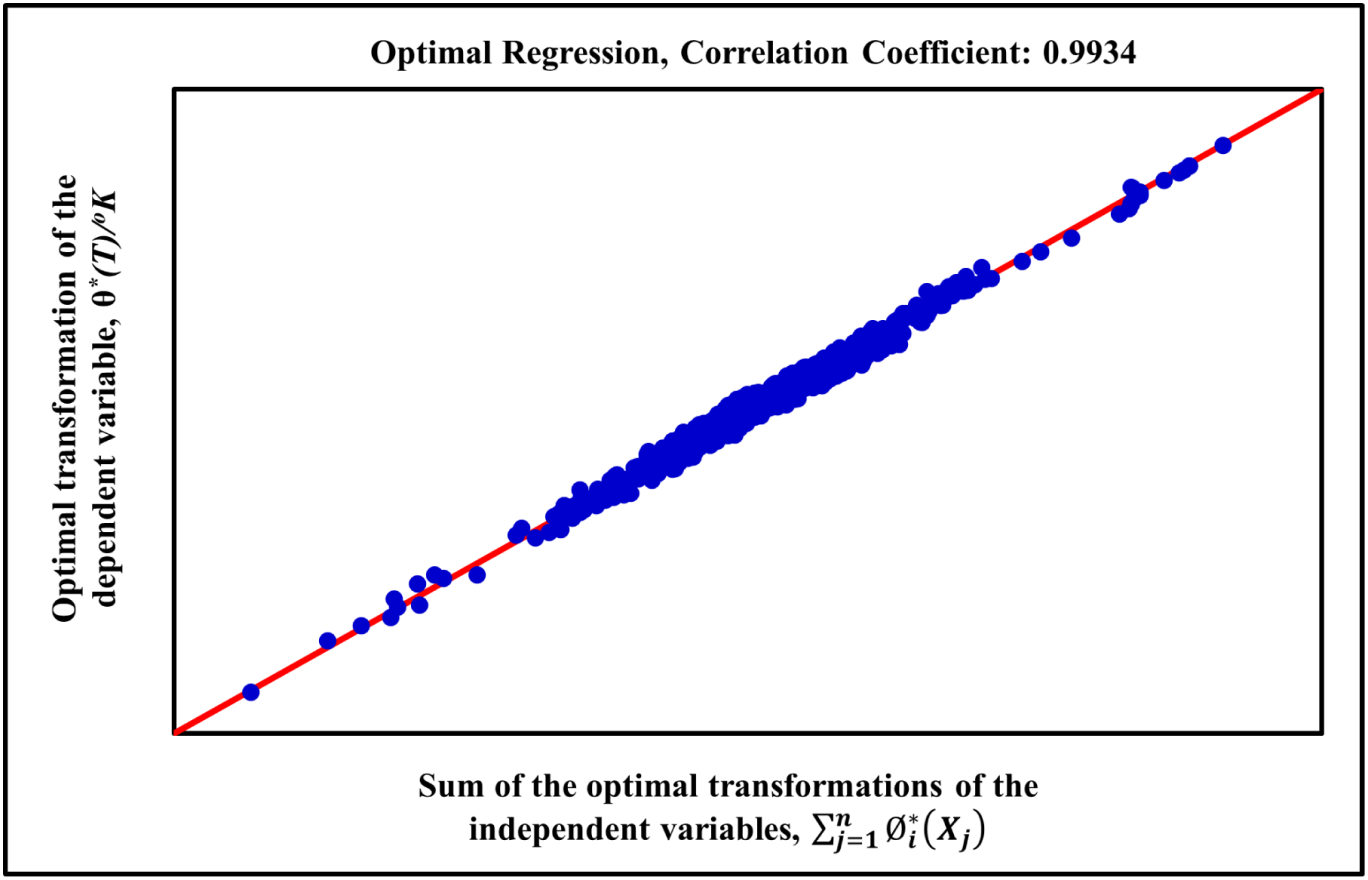
Optimal transformation of the hydrate formation temperature dependent variable, θ*­(*Y*), versus sum of the optimal transformations of the independent variables, $\overset{n}{\underset{j=1}{\sum }}\phi_{j}^{*}\left(X_{j}\right)$ , for the new generalized model.

Figure 2 shows the experimental measurements of the hydrate formation temperature (HFT) versus the resulting inverse of the optimal transformation of the generalized HFT dependent variable, whereas the inverse optimal transformation yielded a final generalized HFT model in the following form:

7 $$\ln\left(T\right)=6.4277\times 10^{-6}Z^{5}+2.4055\times 10^{-5}Z^{4}-1.3462\times 10^{-4}Z^{3}-5.9135\times 10^{-4}Z^{2}+4.0607\times 10^{-2}Z+5.639$$

where

8 $$Z=\overset{20}{\underset{i=1}{\sum }}Z_{i}$$

and

9 $$Z_{i}=A_{5,i}X_{i}^{5}+A_{4,i}X_{i}^{4}+A_{3,i}X_{i}^{3}+A_{2,i}X_{i}^{2}+A_{1,i}X_{i}+A_{0,i}$$

where *A*_5,*i*_, *A*_4,*i*_, *A*_3,*i*_, *A*_2,*i*_, *A*_1,*i*_, and *A*_0,*i*_ are the resulting coefficients of the optimal transformation of each independent variable and listed in Table 2.

**Table 2:** Resulting Coefficients for All the Input Variables

n	X	*A*_5_	*A*_4_	*A*_3_	*A*_2_	*A*_1_	*A*_0_
1	ln(*p*) (kPa)	5.3366 × 10^–5^	–2.6515 × 10^–3^	5.1122 × 10^–2^	–4.6197 × 10^–1^	2.5153 × 10^00^	–7.7564 × 10^00^
2	Molecular weight (g/mol)	–4.9237 × 10^–8^	7.0843 × 10^–6^	–3.6218 × 10^–4^	7.5795 × 10^–3^	1.5080 × 10^–1^	–5.6864 × 10^00^
Hydrocarbon components (mole fraction)							
3	CH_4_	4.5950 × 10^–1^	–5.9034 × 10^00^	8.2808 × 10^00^	–3.6721 × 10^00^	3.5644 × 10^00^	–1.5210 × 10^00^
4	C_2_H_6_	–3.5680 × 10^00^	9.1981 × 10^00^	–8.4845 × 10^00^	3.3676 × 10^00^	1.7699 × 10^–1^	–5.0128 × 10^–2^
5	C_3_H_8_	8.2502 × 10^00^	–2.2287 × 10^01^	2.1756 × 10^01^	–9.2618 × 10^00^	–1.8957 × 10^–1^	9.1809 × 10^–2^
6	i-C_4_H_10_	7.5491 × 10^00^	–1.7275 × 10^01^	1.5601 × 10^01^	–7.2074 × 10^00^	–2.3344 × 10^00^	6.5180 × 10^–2^
7	n-C_4_H_10_	–5.7499 × 10^04^	1.9931 × 10^04^	–2.4119 × 10^03^	1.2315 × 10^02^	–9.6998 × 10^00^	3.8311 × 10^–2^
8	C_5_H_12_	1.0138 × 10^08^	–4.4934 × 10^06^	5.6502 × 10^04^	3.6141 × 10^00^	–1.4841 × 10^01^	6.2719 × 10^–3^
Nonhydrocarbon components (mole fraction)							
9	N_2_	4.9958 × 10^00^	–1.2921 × 10^01^	1.1876 × 10^01^	–4.5763 × 10^00^	7.6689 × 10^–1^	–4.7458 × 10^–3^
10	CO_2_	5.6896 × 10^–1^	–3.9447 × 10^00^	5.6859 × 10^00^	–2.9973 × 10^00^	–2.0519 × 10^00^	7.2354 × 10^–1^
11	H_2_S	–2.6713 × 10^01^	4.5310 × 10^01^	–2.3151 × 10^01^	5.0124 × 10^00^	8.5465 × 10^–1^	–1.7162 × 10^–2^
Salts (mass fraction)							
12	NaCl	–2.5839 × 10^02^	1.6166 × 10^02^	–3.8771 × 10^01^	4.4264 × 10^00^	–5.9585 × 10^00^	2.1672 × 10^–2^
13	MgCl_2_	0.0	7.8200 × 10^01^	–2.6578 × 10^01^	3.0711 × 10^00^	–7.0631 × 10^00^	9.9011 × 10^–3^
14	KCl	0.0	0.0	0.0	3.7000 × 10^–3^	–4.0098 × 10^00^	2.0896 × 10^–3^
Organic inhibitors (mass fraction)							
15	CH_4_O	1.7939 × 10^02^	–1.5270 × 10^02^	3.1926 × 10^01^	–1.9580 × 10^00^	–6.0060 × 10^00^	1.5387 × 10^–1^
16	C_3_H_6_O	–6.7715 × 10^01^	9.8868 × 10^01^	–4.5005 × 10^01^	7.0767 × 10^00^	–1.4243 × 10^00^	7.2744 × 10^–3^
17	C_2_H_6_O_2_	–5.3028 × 10^00^	7.0805 × 10^00^	–3.7778 × 10^00^	1.0856 × 10^00^	–4.2966 × 10^00^	4.6841 × 10^–2^
18	C_3_H_8_O	0.0	0.0	–2.5944 × 10^–1^	1.3333 × 10^–1^	–5.5121 × 10^–1^	1.1175 × 10^–3^
19	C_5_H_9_NO	0.0	0.0	0.0	4.5224 × 10^–2^	–3.4780 × 10^00^	7.6305 × 10^–3^
20	C_3_H_7_NO	0.0	0.0	–4.2818 × 10^–1^	2.7834 × 10^–1^	–4.0415 × 10^00^	1.6626 × 10^–2^

**Figure 2:**
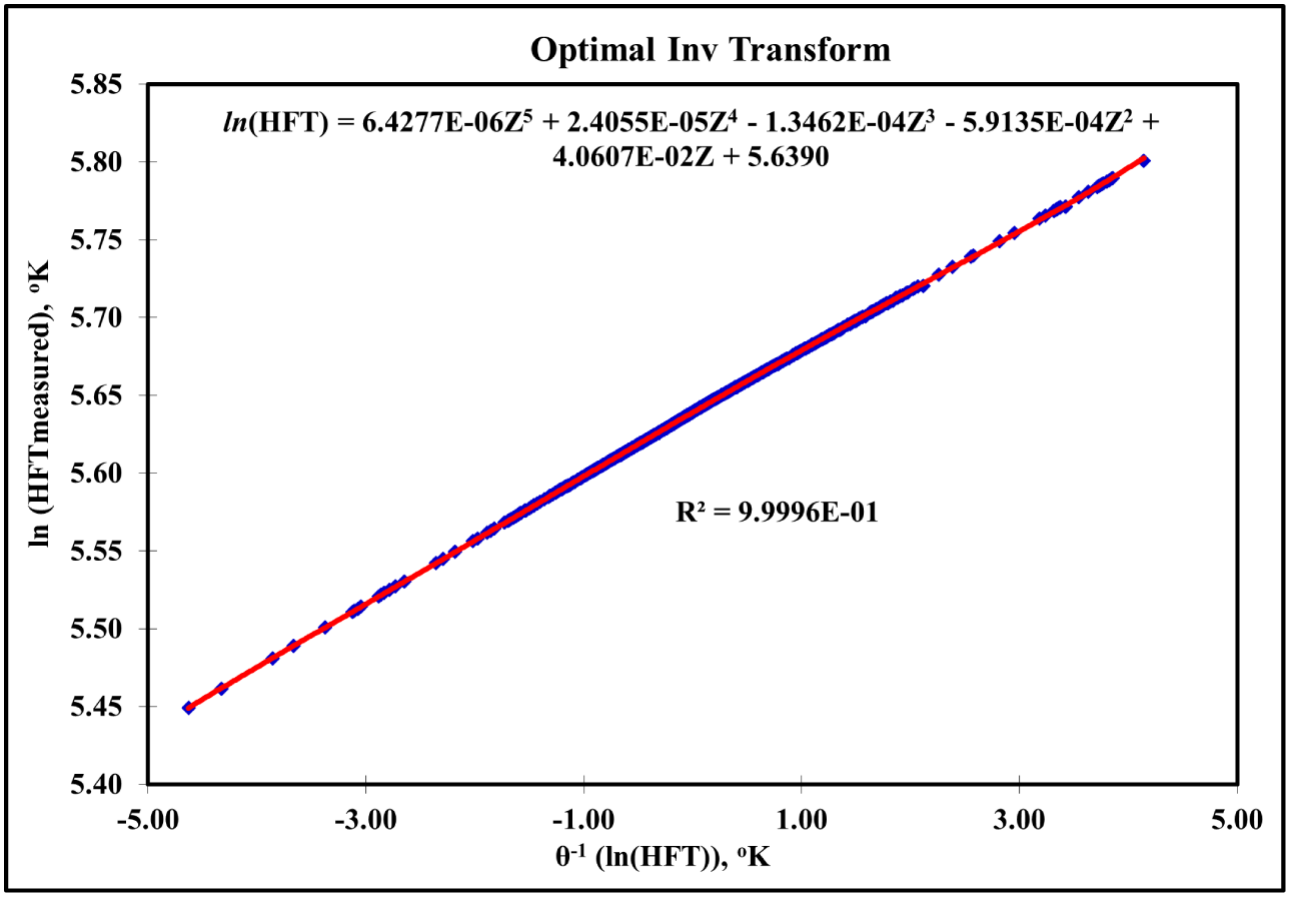
Experimental HFT versus the resulting inverse of the optimal transformation of the generalized HFT dependent variable.

### 3.2 Validation of the New Generalized HFT Model

As mentioned before, about 450 experimental data points were randomly selected from the collected data set for blind testing the developed new model and for comparing its results with the early published correlations. Figure 3 shows a perfect match between the predicted HFT values from the new generalized HFT model and the experimental values for CO_2_, H_2_S, hydrocarbon gases, sweet gas mixtures, and sour gas mixtures in the presence of different salts and organic inhibitors, and/or mixed salts with organic inhibitors. The maximum average relative error (MARE), average absolute relative error (AARE), and the standard deviation (SD) of error for the new model are 1.107%, 0.40%, and 1.378, respectively. For comparison, the model’s overall AARE on the blind test set is 0.40%. For mixed inhibitor systems, this value rises slightly to 0.45%, owing to the increased complexity and synergistic interactions among inhibitors. Nevertheless, this level of accuracy remains satisfactory for industrial design purposes.

**Figure 3:**
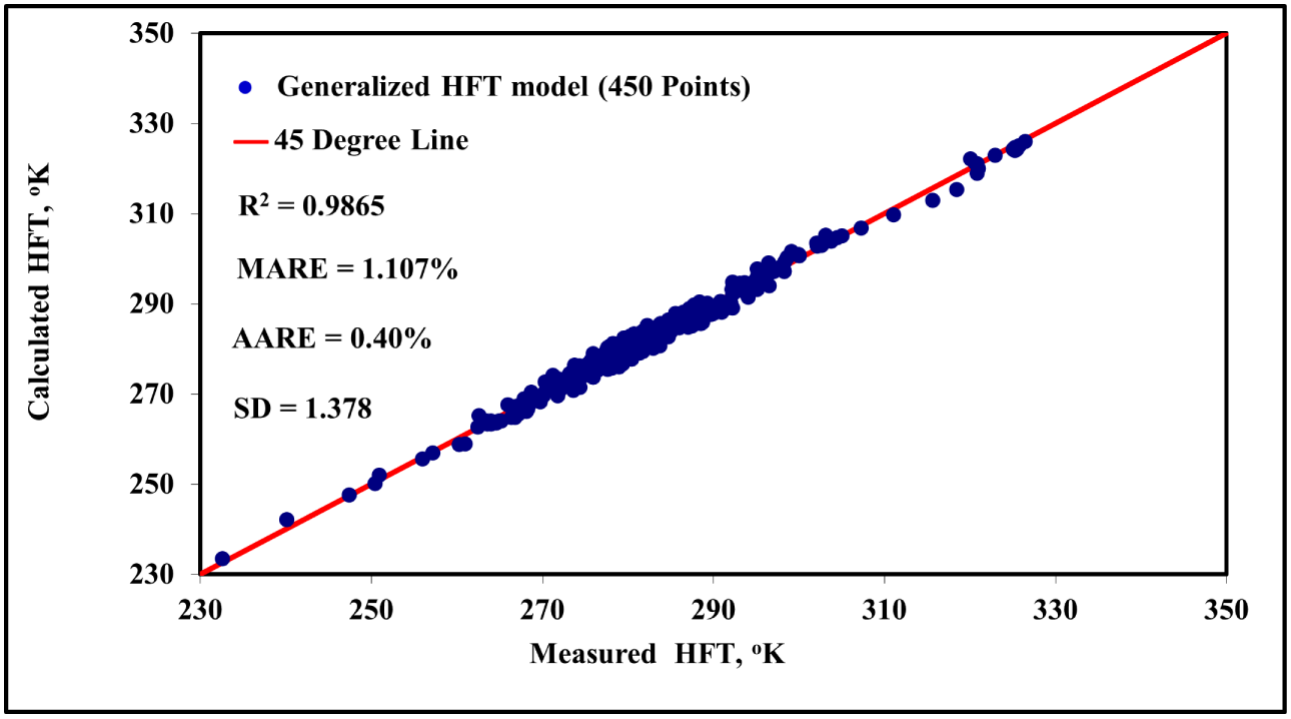
Predicted HFT values from the new generalized HFTmodel and theexperimental values for Co_2_,H_2_S, hydrocarbon gases, sweet gas mixtures, and sour gas mixtures in the presence of different salts and organic inhibitors, and/or mixed salts with organic inhibitors.

Also, Figure 4 shows a perfect match between the predicted HFT values from the new generalized HFT model and the experimental values for CO_2_, H_2_S, hydrocarbon gases, sweet gas mixtures, and sour gas mixtures in the absence of salts and organic inhibitors. The maximum average relative error (MARE), average absolute relative error (AARE), and the standard deviation (SD) of error for the new model are 1.08%, 0.41%, and 1.42, respectively.

**Figure 4:**
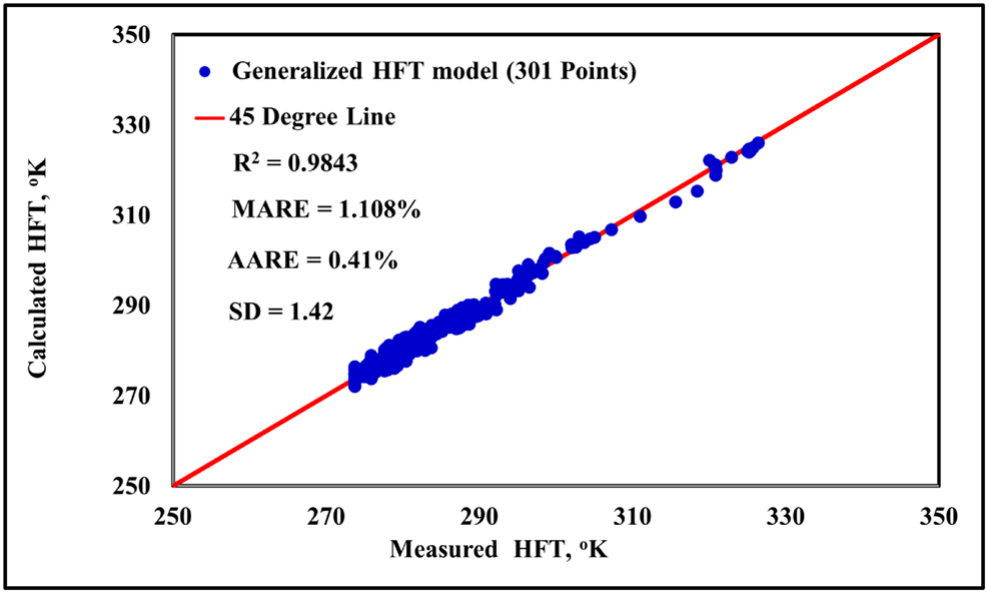
Predicted HFT values from the new generalized model versus the experimental measurements, for CO_2_, H_2_S, hydrocarbon gases, sweet gas mixtures, and sour gas mixtures in the absence of salts and organic inhibitors.

Therefore, the new generalized model can be used to estimate the HFT for CO_2_, H_2_S, pure hydrocarbon gases, sweet gas mixtures, or sour gas mixtures in the presence or absence of different salts and organic inhibitors, and/or mixed salts with organic inhibitors. However, the early published mathematical HFT models are limited to pure hydrocarbon gases, sweet gas mixtures, or sour gas mixtures with very low concentrations of H_2_S and CO_2_ in the absence of hydrate inhibitors. Therefore, the new model was compared with the experimental data and those early published mathematical HFT models.

To compare the new model with the published models, it is recommended to use the same data points based on the limitations of each model. Therefore, the test data set was filtered based on the limitations of each published model. Table 3 summarizes the ranges of the input and output variables for those models. After filtering the test data based on the restriction of each published model (Kobayashi et al.,^16^ Towler and Mokhatab,^17^ Safamirzaei and Namvaran,^20^ Sadeq et al.,^21^ Motiee,^22^ Salufu and Nwakwo,^23^ Ghayyem et al.,^24^ Ghiasi,^25^ Amar^32^ the results of the comparison of those models with the new model using the corresponding data for each model were presented in Figure 5 and Table 4. This comparison showed that the new model is superior to the early HFT published mathematical models for hydrocarbon gases, sweet gas mixtures, and sour gas mixtures.

**Table 3:** Ranges of the Input and Output Variables for Early Published HFT Models[Table-fn tbl3fn1]

Variable	Amar[Bibr ref32] 2021	Sadeq et al.[Bibr ref21] 2018	Safamirzaei and Namvaran[Bibr ref20] 2015	Salufu and Nwakwo[Bibr ref23] 2013	Ghayyem et al.[Bibr ref24] 2014	Ghiasi[Bibr ref25] 2012	Towler and Mokhatab[Bibr ref17] 2005	Motiee[Bibr ref22] 1991	Kobayashi et al.[Bibr ref16] 1987
*T*/K	273.7–286	273–330	273–330	273–299	273–299	272–299	a	>289	274–289
*P*/kPa	582–6285.0	1.7–330	0.591–62.011	0.35–30.0	0.35–30.0	0.37–30.0	a	0.58–86.8	>14.0
Molecular weight/g mol^–1^	16.22–24.18	16.02–28.97	16.8–23.172	16–29	16–29	15.642–29	16.04–44.1	<18.83	15.99–26.07
Hydrocarbon components (mole fraction)									
CH_4^*^_	b	Pure gases and gas-mixtures without H_2_S	0.654–0.965	Pure and mixture of hydrocarbon systems only					
C_2_H_6^*^_	b		0.009–0.127						
C_3_H_8^*^_	b		0.00–0.103						
i-C_4_H_10^*^_	b		0.0–0.0099						
n-C_4_H_10^*^_	b		0.0–0.037						
C_5_H_12^*^_	0.0–0.035		0.0–0.0101						
N_2^*^_	b		0.0–0.15						
CO_2^*^_	0.0–0.25		0.0–0.0325						
H_2_S^*^	0.0–0.27		0.0–0.0025						
C_1_ + C_2^*^_	0.66–0.99		b						
C_3_ + C_4_ + N^*^	0.0–0.281		b						

i Mole fraction. a: no limitation. b: not used alone.

**Table 4:** Comparison between Early Published HFT Models and the New Generalized Model over Test Dataset Limited According to the Restrictions of Each Model for Hydrocarbon Gases, Sweet Gas Mixtures, and Sour Gas Mixtures without Inhibitors

Model	*R* ^2^	AARE (%)	MARE (%)	SD	No. of points
New generalized model^a^	0.9843	0.41	1.108	1.42	301
Amar, 2021 ^32^	0.958	0.469	2.85	2.17	107
Sadeq et al., 2018 ^21^	0.926	1.26	4.998	5.69	113
Safamirzaei & Namvaran, 2015 ^20^	0.988	0.42	1.279	1.65	35
Salufu & Nwakwo, 2013 ^23^	0.88	0.844	2.2	2.91	44
Ghayyem et al., 2014 ^24^	0.962	0.852	2.51	3.1	79
Ghiasi, 2012 ^25^	0.833	1.28	2.64	4.21	44
Towler & Mokhatab, 2005 ^17^	0.983	1.185	2.76	3.91	86
Motiee, 1991 ^22^	0.963	2.13	5.57	9.35	42
Kobayashi et al., 1987 ^16^	0.77	4.03	8.95	13.37	21

a Contain the same data points in all the compared models based on the data ranges in Table 1 without inhibitors.

**Figure 5:**
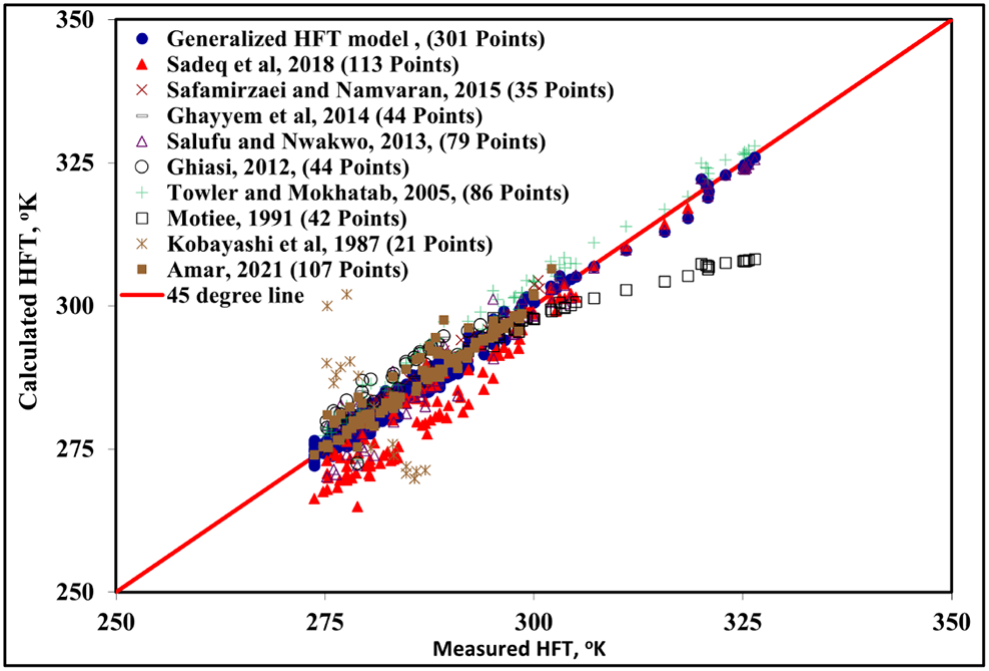
Predicted HFT values from the new generalized model versus the experimental measurements, for CO_2_, H_2_S, hydrocarbon gases, sweet gas mixtures, and sour gas mixtures in the presence of different salts and organic inhibitors.

## 4 Discussion

The development of a generalized hydrate formation temperature (HFT) model using the Alternating Conditional Expectation (ACE) algorithm marks a significant step forward in correlative prediction for gas hydrate management. The model’s robust performance, demonstrated by its low error metrics (AARE ∼ 0.4%) across a vast and diverse blind-test data set, invites a deeper analysis of the factors driving its success, the insights gleaned from its structure, and the boundaries of its application.

### 4.1 Superior Performance of the ACE Algorithm: Handling Complexity and Nonlinearity

The demonstrated superiority of the present ACE-based model, as shown in Table 4 and Figure 5, can be directly attributed to its ability to navigate the intricate, nonlinear physical landscape of hydrate formation. Conventional empirical correlations^16,17,22^ rely on fixed mathematical forms, such as linear or logarithmic relationships with gas gravity or pressure. While useful for narrow application windows, these predefined forms are fundamentally constrained in their capacity to represent the complex, interdependent effects of gas composition and thermodynamic inhibition.

The ACE algorithm transcends this limitation by not assuming a relationship, but rather by discovering one. Its core mechanism involves finding optimal transformations for both the predictors and the response variable to create a space where their relationship is maximally linear. The striking linear trend in Figure 1, plotting the transformed HFT against the sum of the transformed predictors, is the most direct evidence of this success. This plot is more than a goodness-of-fit indicator; it is a visual testament that the complex, multidimensional physics governing hydrate equilibrium can be effectively linearized through a sophisticated, data-driven rescaling of the variables. This allows the model to accurately capture the nonclassical, concentration-dependent depression of the HFT caused by various salts and organic inhibitors, effects that are poorly represented by simple, additive terms in traditional correlations.

Furthermore, the model’s granular approach of applying separate transformations to individual gas components (e.g., ϕ*­(CH_4_), ϕ*­(CO_2_)) provides a critical advantage over methods using lumped parameters like gas gravity. A single gravity value cannot distinguish the distinct hydrate-forming potentials of different gas molecules. By treating each component independently, the ACE model can implicitly account for their unique roles in stabilizing the hydrate lattice, leading to more accurate predictions for complex sweet and sour gas mixtures.

### 4.2 Interpreting the Model’s Architecture and Output

The final predictive tool, encapsulated in eqs 7–9, is highly effective but requires a nuanced understanding. The variable *Z* represents the aggregate signal from all the transformed input variables. eq 7, a high-order polynomial linking *Z* to the natural logarithm of temperature, acts as the “decoder” that translates the linear relationship from the transformed space back into a usable temperature prediction in the original physical domain.

Figure 2 serves as the ultimate validation of this decoding process. The excellent agreement between the experimental HFT and the model’s output, evident from the tight data scatter along the 45-degree line across a broad temperature spectrum, confirms the fidelity of the entire transformation sequence. The consistently high *R* ^2^ values reported in Figure 3 (for systems with inhibitors) and Figure 4 (for systems without inhibitors) further cement the model’s reliability and its general applicability across different system conditions.

A critical point of discussion is the physical interpretability of the individual $\phi_{i}^{*}\left(X_{i}\right)$ transformations. These transformations, defined by the polynomial coefficients in Table 2, are statistical constructs optimized for predictive power within the ACE framework. They should not be misinterpreted as direct representations of thermodynamic properties, such as chemical potential or Langmuir constants. For example, the complex fifth-order polynomial for methane does not describe its affinity for specific hydrate cages. Instead, it functions as a sophisticated scaling factor that, when combined with all other component transformations, produces the correct collective impact on the HFT. This statistical nature is a trade-off, sacrificing some physical transparency for the considerable gain in predictive accuracy and generality across a wide parameter space.

### 4.3 Comparative Analysis with Published Models

The comparative analysis detailed in Figure 5 and Table 4 provides unambiguous evidence of the new model’s enhanced capability. When evaluated on data sets conforming to the limitations of earlier models, our model consistently delivers the best performance, with the lowest AARE and standard deviation.

Simpler, older correlations like those of Kobayashi et al.^16^ and Motiee^22^ exhibit significantly higher error rates, a predictable outcome given their inability to model the complex nonlinearities that the ACE algorithm handles effectively. More contemporary models, such as those by Sadeq et al.^21^ and Ghiasi,^25^ show improved accuracy but are still outperformed. This is likely due to their more constrained scope, often excluding the effects of inhibitors or specific “sour” components like H_2_S, which our model explicitly includes.

It is particularly telling that our generalized model achieves accuracy comparable to, or even surpassing, that of Safamirzaei and Namvaran^20^ on its own restricted data set. This indicates that the model’s broad applicability does not come at the cost of precision in more conventional scenarios. This collective evidence positions the ACE-based model not as a minor incremental update, but as a substantive advancement that consolidates the predictive scope of numerous predecessor models into a single, more universally applicable framework.

Furthermore, to establish a comprehensive benchmark for the proposed ACE-based model, its predictive performance was compared against two widely accepted thermodynamic frameworks: the van der Waals–Platteeuw^50^ (vdW-P) model and the Chen–Guo^51^ model. The comparison was conducted using the same blind test data set (450 experimental data points), that covering a broad range of gas compositions, pressures, temperatures, and different inhibitor conditions. The vdW-P model was implemented with the Peng–Robinson equation of state and the Parrish–Prausnitz^7^ correlation for Langmuir constants, while the Chen–Guo model was implemented with the Patel-Teja equation of state. For mixed inhibitor systems (salt + organic inhibitor), the Chen–Guo model was coupled with the N-NRTL-NRF^52^ activity model. Chen–Guo^51^ and vdW–P thermodynamic models yielded AARE of 2.85% and 3.21% respectively. However, both models exhibited elevated errors for systems containing mixed inhibitors and for sour gas mixtures with high H_2_S concentrations. In contrast, the proposed ACE–based model substantially reduced prediction errors across all tested conditions. This comparative analysis clarifies that the proposed ACE–based model is not intended to replace thermodynamic models. Rather, it offers an alternative tool that prioritizes computational speed and ease of use while maintaining high accuracy within its validated domain.

### 4.4 Model Limitations and Scope for Application

The model’s validity is confined to the ranges of the training data, as comprehensively outlined in Table 1. Employing the model outside these boundaries—for instance, at pressures exceeding 330 MPa, with inhibitor types or concentrations not represented in the data set, or for gas compositions with molecular weights beyond the specified range—carries a high risk of erroneous predictions. The high-order polynomials are particularly prone to unphysical behavior when extrapolated.

## 5 Conclusions

The ACE-based HFT model presented here serves as a highly accurate and comprehensive correlative tool for industrial design and risk assessment. Its development highlights the power of nonparametric regression techniques to overcome the limitations of traditional empirical correlations when applied to complex physical systems with multiple interacting variables. Based on the results of this new model, the following conclusions are drawn:

(1) The new HFT model yields a perfect match with the experimental measurements of the HFT for CO_2_, H_2_S, pure hydrocarbon gases, sweet gas mixtures, and sour gas mixtures in the presence of different salts and organic inhibitors.
(2) The new model is superior to the early HFT published mathematical models for hydrocarbon gases, sweet gas mixtures, and sour gas mixtures without inhibitors.
(3) This model covers a wide pressure range of 1.7–330 MPa, temperature range of 273–320 K, gas mixture molecular weight range of 16.04–55.6, and a wide range of gas mixture components and inhibitors.
(4) This model is a powerful tool for estimating the HFT for CO_2_, H_2_S, pure hydrocarbon gases, sweet gas mixtures, and sour gas mixtures in the presence of different salts and organic inhibitors in the lack of experimental measurement.

## Nomenclature

HFT: Hydrate formation temperature
*X*_1_, *X*_2_,···, *X*_*p*_: Independent or predictor variables
*Y*: Dependent or response variable
*e**: ACE regression error
E: Mathematical expectation
θ­(*Y*): Transformation for dependent variable
θ*­(*Y*): Optimal transformation for dependent variable
ϕ_*i*_(*X*_*i*_): Transformation for independent variable
$\phi_{i}^{*}\left(X_{i}\right)$: Optimal transformation for independent variable
**Average relative error, ARE, %**
AARE: Average absolute relative error, % $\mathrm{AARE}=\frac{100}{N}\overset{N}{\underset{k=1}{\sum }}\left|\frac{{\mathrm{HFT}}_{k,\mathrm{calc}}-{\mathrm{HFT}}_{k,\mathrm{meas}}}{{\mathrm{HFT}}_{k,\mathrm{meas}}}\right|$
MARE: Maximum absolute relative error, % $\mathrm{MARE}=\underset{k}{\max}\left|\frac{{\mathrm{HFT}}_{k,\mathrm{calc}}-{\mathrm{HFT}}_{k,\mathrm{meas}}}{{\mathrm{HFT}}_{k,\mathrm{meas}}}\right|\times 100$
SD: Standard deviation $\mathrm{SD}=\sqrt{\frac{\underset{k}{\sum }{\left({\mathrm{HFT}}_{k,\mathrm{calc}}-{\mathrm{HFT}}_{k,\mathrm{meas}}\right)}^{2}}{N-1}}$
